# Interferon-γ Release Assay Control Results Are Associated With All-Cause Mortality

**DOI:** 10.1093/geroni/igaf122.3611

**Published:** 2025-12-31

**Authors:** Benjamin Seligman, David Ganz, Matthew Goetz, David Beenhouwer

**Affiliations:** UCLA School of Medicine, Los Angeles, California, United States; VA Greater Los Angeles Healthcare System, Los Angeles, California, United States; VA Greater Los Angeles Healthcare System, Los Angeles, California, United States; University of California, Los Angeles, Los Angeles, California, United States

## Abstract

Impaired immune responses are a key feature of aging. However, there are no clinical tests of immune function. Interferon-gamma release assays (IGRAs) for tuberculosis screening may provide such a measure. We assess this measure’s relationship with all-cause mortality. IGRAs quantify release of interferon-gamma by T-cells and the difference between unstimulated and mitogen-stimulated T-cells is assessed for test validity. This mitogen-nil difference captures T-cell function. We obtained all negative and indeterminate IGRA tests from a large health system along with demographics, frailty, and area deprivation index (ADI) data. We limited our sample to the most recent test given to outpatients not receiving hemodialysis or immunosuppressive medications. We assessed the association of the mitogen-nil difference with mortality at 6 months, 1 year, and 5 years by Cox regression. Among 16,105 unique individuals, reported mitogen-nil results ranged from < 0.01 to > 10 IU/mL. Relative to values from 0-1, values >10 had HRs (95%) for mortality of 0.33 (0.18 - 0.63), 0.41 (0.26 - 0.65), and 0.52 (0.39 - 0.70) at 6 months, 1 year, and 5 years respectively after adjustment for age, sex, and test result (indeterminate versus negative). Including frailty, HRs were 0.36 (0.19 - 0.68), 0.45 (0.29 - 0.70), and 0.57 (0.42 - 0.77). With frailty and ADI, HRs were 0.41 (0.20 - 0.83), 0.50 (0.30 - 0.84), 0.60 (0.43 - 0.83). Greater T-cell response to mitogen stimulation in IGRAs is associated with lower hazard of mortality. This common, commercially available lab test may provide additional information to risk-stratify patients.

